# How to prevent endophthalmitis after intravitreal injections

**DOI:** 10.1186/s40942-015-0012-9

**Published:** 2015-08-11

**Authors:** Roger A Goldberg

**Affiliations:** Bay Area Retina Associates, Walnut Creek, CA 94598 USA

**Keywords:** Intravitreal injection, Endophthalmitis, Antisepsis

## Abstract

Endophthalmitis is an uncommon but often visually devastating complication of intravitreal injections. This commentary reviews the key aspects and technical components of intravitreal injections and how they may relate to the development of endophthalmitis. Because endophthalmitis is a rare event, data are often lacking on specific aspects of injection technique that may or may not be helpful in preventing infection. What is not in doubt, however, is the utmost importance of using povidone-iodine antisepsis to the ocular surface prior to injection, and maintaining a lash and lid margin-free injection site.

## Background

Intravitreal injections are routinely performed in a busy vitreoretinal clinic. They are generally safe, but their feared complication—endophthalmitis—can be a visually devastating event. Understanding the key steps to the injection procedure may help minimize the risk of endophthalmitis, and perhaps drive the event rate lower than it currently stands. The aim of this Commentary is to discuss some of these key steps.

## Main text

Intravitreal drug delivery has revolutionized retinal care over the past decade, driven largely by the skyrocketing use of anti-vascular endothelial growth factor (anti-VEGF) agents for the treatment of neovascular age-related macular degeneration (AMD), retinal vein occlusion (RVO), and diabetic macular edema (DME) [1]. These injections are generally safe and well-tolerated, and effective at stabilizing vision in most patients, and improving vision in some patients. One downside to these drugs is their relatively short duration of effect; patients often need frequent, sometimes monthly, injections to maintain visual gains. It is not an uncommon scene, then, in a busy vitreoretinal clinic, to see patients receiving their 20th, 30th or even 40th injection.

One challenge this need for repeat injections leads to is an increased likelihood of a patient experiencing a complication of an intravitreal injection over the lifetime of their treatment. The most feared complication of intravitreal injection is endophthalmitis, which can be visually devastating to the affected eye. The rate of endophthalmitis is somewhere between 1 and 2,000–5,000 injections [2], far less than the 1 in 400 rate of post-operative endopthalmitis seen after cataract surgery [3]. However, unlike cataract surgery, which a patient experiences only one time (per eye), an intravitreal injection is a frequently-repeated process, which can transform a low *per injection* complication rate into a higher *per patient* complication rate.

Though longer-acting agents may be on the horizon, intravitreal injections will likely be a key component of retinal care for the foreseeable future. Given this reality, how can we, as vitreoretinal specialists, drive the rate of endophthalmitis after intravitreal injection even lower? One prerequisite to answering this question is to fully understand where we are and what we know now. How frequently does infection occur? What are the common causative organisms? What are the critical steps to the procedure? What techniques have been shows to be effective—or not effective—at reducing the risk of endophthalmitis?

To this end, Drs. Merani and Hunyor [4] took an important step to answer these questions by summarizing and synthesizing the critical data and expert opinions on endophthalmitis after intravitreal injections. They limit their literature review to published articles on endophthalmitis after intravitreal anti-VEGF injections, and exclude data from clinical trials or from those that include steroid or other non-anti-VEGF injections. Both of these are reasonable decisions. Clinical trials often exclude certain patient populations or mandate protocol-specific injection techniques that may not mimic what happens every day in vitreoretinal clinics. Similarly, the rate of endophthalmitis after intravitreal steroid injections has been reported to be higher than that seen after intravitreal anti-VEGF injections [5], which may be related to the drug, drug vehicle, or patient population and disease states requiring steroid injections.

The authors identified 20 papers that met their inclusion criteria, with each case series having at least 10,000 injections. Over 500,000 injections were pooled, and 144 cases of endophthalmitis were identified. This leads to a rate of 0.028 % [4], which is consistent with previously reported rates.

The reported incidence of endophthalmitis appears to be marginally lower in an operating room-based setting (a common location for intravitreal injections in Europe) versus an office location (where the vast majority of injections occur in the United States) [4]. However, these studies were all retrospective in nature, and it can be difficult to compare the different series. Additionally, the logistical challenges and extensive cost that would be required to shift office-based injections to an operating room would be impractical, at least in the United States.

Would mimicking the operating room environment in the clinic help? In several instances, Merani and Hunyor suggest this may be helpful, though without sufficient evidence to support their recommendation. For example, they suggest that sterile gloves should be worn by the treating clinician, though acknowledge no data exist as to whether gloves at all—let alone sterile gloves—help reduce the rate of endophthalmitis [4]. Similarly, while Wen et al. demonstrated some laboratory simulations of the effect of talking on bacterial growth [6], the use of facemasks specifically, whether worn by the injecting clinician, the technician, or the patient, has not been shown to lower the risk of endophathalmitis. Limiting talking during the procedure may be sufficient.

No data regarding hand antisepsis exists to support one type of hand antisepsis over another, though maintaining good hand hygiene, including the use of alcholol-based agents before and after patient contact, seems to be a good idea. This is the current recommendation for every patient encounter, not just for those receiving intravitreal injections [7].

The package inserts for both ranibizumab (Lucentis, Genentech/Roche, South San Francisco, CA) and aflibercept (Eylea, Regeneron, Tarrytown, NY) state that sterile gloves, drape, and speculum should be used with each injection [8, 9]. Here again, a paucity of useful data is available to guide us as to whether these three items can help reduce endophthalmitis. Certainly, adequate hand hygiene is required, as is sufficient retraction of the lid margin from the injection site. The lid speculum is often uncomfortable for patients, and manual retraction of the lids is often sufficient to achieve the goal of avoiding eyelid contact with the intended injection site and needle [10].

Merani and Hunyor recommend disinfecting the rubber diaphragm of ranibizumab or aflibercept vials with an alcohol swab immediately before drawing up the medicine into the tuberculin syringe [4]. However, if the metal cap is removed immediately prior to puncturing the vial, with care being taken to avoid touching the rubber diaphragm, this step may be unnecessary, or even harmful. Numerous instances of contaminated alcohol prep pads and swabs have been reported, in some cases leading to nationwide recalls [11].

Ultimately, what is not debated is the paramount importance of topical antisepsis prior to intravitreal injection. For this, povidone iodine solutions are the gold standard. Standard surgical skin preparations are typically 10 % povidone iodine concentration, and include detergents that are toxic and irritating to the ocular surface. Betadine (Alcon Laboratories, Fort Worth, Tx) is a 5 % povidone iodine solution designed for ocular surface anti-sespsis. It can still be irritating and uncomfortable to the ocular surface, even with a pre-treatment anesthetic drop such as proparacaine. It should be applied before any viscous anesthetic gels to achieve adequate antisepsis. Pre- or post-injection antibiotics do not help prevent endophthalmits, and may be harmful by inducing microbial resistance [10].

## Conclusion

Given the projected growth of intravitreal injections [1], we must continue to search for new and better ways to further reduce the rate of endophthalmitis after each injection. Doing so may enable us to drive the *per patient* rate of infection lower than where it stands today. Though endophthalmitis after intravitreal injection may not be a “never event,” [12] we must still strive to do better.

## Abbreviations

Anti-VEGF: anti-vascular endothelial growth factor
AMD: neovascular age-related macular degeneration
RVO: retinal vein occlusion
DME: diabetic macular edema
